# Lactylation‐Related Genes in Ulcerative Colitis: A Multiomics Mendelian Randomization Study for Therapeutic Target Discovery

**DOI:** 10.1155/humu/7720538

**Published:** 2026-06-26

**Authors:** Na An, Ruiyun Wang, Huiping Jiao, Zhongwen Lu, Yafei Dou, Laikun Zheng, Zhaoliang Ding

**Affiliations:** ^1^ Anorectal Department, Shandong University of Traditional Chinese Medicine, Jinan, Shandong, China, sdutcm.edu.cn; ^2^ Nursing Department, Rizhao Hospital of Traditional Chinese Medicine, Rizhao, Shandong, China, rzhtcm.com; ^3^ Education Department, Rizhao Hospital of Traditional Chinese Medicine, Rizhao, Shandong, China, rzhtcm.com; ^4^ Orthopedics Department, Beijing University of Chinese Medicine Third Affiliated Hospital, Beijing, China, bucm.edu.cn; ^5^ Ultrasound Department, Rizhao Hospital of Traditional Chinese Medicine, Rizhao, Shandong, China, rzhtcm.com; ^6^ Anorectal Department, Rizhao Hospital of Traditional Chinese Medicine, Rizhao, Shandong, China, rzhtcm.com

**Keywords:** lactylation, Mendelian randomization, multiomics integration, qPCR, spatial transcriptome, ulcerative colitis

## Abstract

**Background:**

Ulcerative colitis (UC), a chronic and nonspecific intestinal inflammatory disease, has seen a rising incidence rate worldwide year by year. Its pathogenesis remains incompletely understood. Lactylation modification is closely related to the generation of inflammation. Exploring the pathogenesis of UC from the perspective of lactylation is of great significance for the understanding and treatment of UC.

**Method:**

Forty‐six lactylation genes were retrieved from the literature and intersected with the eQTLGen whole blood cis‐eQTL to screen 29 candidate genes. Taking UC (finngen_R12_ULCERNAS, 482,657 participants) in the Finnish database as the outcome, significant eQTLs were selected as instrumental variables. The core genes were identified through multimodel Mendelian randomization with inverse variance weighting and heterogeneity and pleiotropy tests. Subsequently, the causal association was reverified by SMR, and differential expression analysis was performed after batch correction in combination with the GEO dataset. The expression profiles of cell subpopulations were analyzed based on single‐cell data (GSE214695). Finally, the UC inflammation model was constructed by LPS‐induced colonic epithelial cells, and the expression changes of the target genes were verified and determined by qPCR experiments. Then, the expression of the genes in the UC was re‐examined in the spatial transcriptome samples.

**Result:**

Mendelian randomization identified EP300, LDHC, and STMN1 as causal genes involved in the lactylation mechanism of UC. The GEO dataset shows that EP300 and STMN1 are significantly upregulated in the UC group. Single‐cell maps revealed that STMN1 was enriched in B cells, LDHC, and EP300 in endothelial cells. The qPCR experiment and spatial transcriptome sample confirmed that STMN1 was upregulated most significantly.

**Conclusion:**

STMN1 and EP300 have been identified as causal genes for UC. The expression of STMN1 in UC significantly increased, and it was specifically enriched in B cells and monocytes. Lactic acid modification may participate in the pathogenesis of UC by driving immune imbalance, providing a genetic basis for the development of new diagnostic markers and targeted lactate intervention strategies.

## 1. Introduction

**Figure 1:**
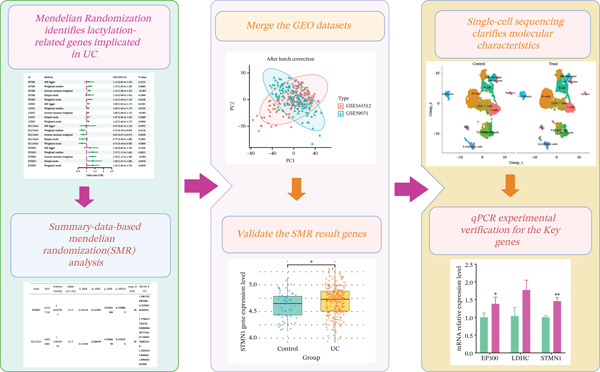
Flowchart of this study.  ^∗^ *p* < 0.05,  ^∗∗^ *p* < 0.01.

Ulcerative colitis (UC) is a chronic, nonspecific intestinal inflammatory disease of unknown cause. Its lesions mainly involve the colonic mucosa and submucosa. The clinical manifestations are persistent or recurrent diarrhea, mucous bloody stools, and abdominal pain. The course of the disease is long and often recurs. The risk of colorectal cancer in patients is significantly increased [1, 2]. Although the exact cause is unknown, it is generally believed to be caused by the combined effects of genetic susceptibility, abnormal responses of the intestinal mucosal immune system, imbalance of the intestinal microbiota, and environmental factors [1]. In recent years, the role of epigenetic modifications in UC has attracted increasing attention. Lactylation is a newly discovered posttranslational modification (PTM) of proteins. It occurs when lactic acid molecules covalently bind to lysine residues in proteins via the metabolic intermediate lactoyl‐CoA (lactyl‐CoA), forming a lactoyl modification. This modification can alter the conformation and function of proteins, thereby regulating gene expression, metabolic reprogramming, and the tumor microenvironment [3, 4]. In recent years, various omics and computational biology methods have been widely applied in the exploration of the mechanism of UC, such as the construction of immune‐related gene diagnostic models based on machine learning [5]. Mendelian randomization (MR) leverages genetic variants as instrumental variables for causal inference, effectively overcoming confounding and reverse causation biases inherent in traditional observational studies, thus holding unique value in biomarker discovery [6]. Integrative bioinformatics and machine learning approaches enable systematic dissection of transcriptomic features and key regulatory networks, promising the identification of novel biomarkers with diagnostic and prognostic value [7]. Further integration of single‐cell transcriptomics with MR allows the identification of causal biomarkers from multiomics data, significantly enhancing discovery reliability by addressing confounding and reverse causation [8]. MR combined with multiomics methods can accurately screen causal biomarkers from large‐scale data, providing more reliable molecular targets for disease diagnosis and treatment. However, systematic research on genes related to lactic acid modification in UC is still relatively scarce. This study innovatively combines lactic acid modification with the genetic susceptibility of UC. We use MR to avoid confounding bias common in traditional observational studies. This approach screens lactic acid–related genes that may have causal relationships with UC. Furthermore, multilevel verification is conducted through multiomics data and experiments. These findings provide new insights into the mechanisms and precise treatment of UC. The research workflow is illustrated in Figure 1.

## 2. Method

### 2.1. Lactylation‐Related Gene Screening and Expression Quantitative Trait Loci (eQTL) Data Acquisition

A total of 46 genes related to lactylation that have been experimentally verified were systematically collected and sorted out from existing scientific literature [9–12]. The cis‐eQTL summary data (sample size > 30,000) from whole blood was downloaded from the eQTLGen consortium (https://www.eqtlgen.org/), which provides information on the association between single‐nucleotide polymorphisms (SNPs) and gene expression levels. The 46 lactated gene lists were intersected with the genes corresponding to all significant eQTLs in eQTLGen, and ultimately, 29 candidate genes that were both involved in lactated modification and had significant eQTL signals were obtained.

### 2.2. Outcome Data and MR Analysis

This study conducted MR analysis based on three core assumptions: the correlation assumption, which states that the selected SNPs are strongly correlated with the exposure (expression levels of lactylation‐related genes); the independence assumption, which asserts that the SNPs are not associated with confounding factors that affect the exposure–outcome relationship; and the exclusion restriction assumption, which stipulates that the SNPs only influence the outcome (UC) through their effect on the exposure, with no other pathways involved. The genome‐wide association study (GWAS) summary data of UC originated from the FinnGen consortium R12 release, which contains the genetic information of thousands of UC cases and controls. This study employed a two‐sample MR design. The exposure data (eQTL) were obtained from the eQTLGen consortium with predominantly European ancestry, whereas the outcome data (UC GWAS) were from the FinnGen database of the Finnish population. Although the two samples were independent, they both mainly originated from the European population, and their genetic backgrounds were comparable, which reduced the risk of bias caused by population stratification. We noticed that there might be some unknown overlap between the two samples, so we mainly used the random effects inverse variance weighted (IVW) model, which was relatively robust to sample overlap. Specifically, the eQTLGen consortium consists of 31,684 blood and PBMC samples from 37 independent cohorts predominantly of European ancestry, with no Finnish biobank samples included [13]. The FinnGen consortium (Release 9) is a Finnish population‐specific cohort comprising up to 392,396 participants of Finnish ancestry, with samples exclusively collected from Finnish biobanks [14]. Given that eQTLGen contains no Finnish samples and the two datasets originate from geographically and genetically distinct populations, sample overlap between the exposure and outcome datasets is negligible. First, utilizing R 4.5.0 software, along with the TwoSampleMR, pacman, and ieugwasr packages, SNPs from the eQTL data of 29 candidate genes that were significantly correlated with gene expression (*p* < 5 × 10^−8^) and independent of each other (clumping *r* ^2^ < 0.001, window size = 10,000 kb) were selected as instrumental variables. Then, five MR methods were used for estimation: MR‐Egger, weighted median, IVW, simple mode, and weighted mode. Among them, the IVW method serves as the main analytical approach. To evaluate the reliability of MR results, we conducted a sensitivity analysis and used Cochran′s *Q* test to assess the heterogeneity among instrumental variables. The effects of horizontal pleiotropy were evaluated and corrected using the MR‐Egger intercept test and the MR‐PRESSO (Pleiotropy RESidual Sum and Outlier) global test. Analyze and test whether the results are driven by a single potent SNP through the “retention method.” Finally, genes with *p* < 0.05 in the IVW method and passing the polymorphism test (MR‐PRESSO global test *p* > 0.05) were regarded as significant genes. A total of four core genes, namely, EP300, LDHC, SLC16A4, and STMN1, were screened out.

### 2.3. Summary‐Data‐Based Mendelian Randomization (SMR) Analysis and Verification

To further enhance the reliability of the research results and verify the associations between the four previously discovered significant genes and the disease, we adopted the SMR method for further analysis. The SMR method assesses the potential causal impact of gene expression levels on disease risk by integrating data from eQTL and GWASs. One key advantage of this method lies in its ability to identify and eliminate false positive associations caused by linkage disequilibrium (LD), thereby enhancing the accuracy of inference. In this analysis, an association was considered significant if *p*_SMR < 0.05. The HEIDI test was used to distinguish causal effects from LD, and a *p*_HEIDI > 0.05 was considered no significant heterogeneity, supporting a true causal relationship.

### 2.4. Batch Effect Removal Following GEO Dataset Integration

Two datasets of UC (GSE59071 and GSE165512) were obtained from the GEO database. After standardizing the two datasets, the ComBat function from the “sva” package (V3.44.0) in RStudio software was used to correct the batch effect. Merge the two datasets to reduce the genetic variance bias of a single dataset, and then conduct variance analysis on the resulting genes screened out by SMR analysis. We selected three key genes, EP300, LDHC, and STMN1, and plotted box plots of their expression levels, respectively, thereby visually demonstrating the distribution differences and dispersion degrees of these genes between the UC group and the control group.

### 2.5. Single‐Cell Sequencing Analysis

In this study, the GSE231993 dataset was first downloaded from the NCBI GEO database and applied to the exploration of expression correlation at the single‐cell level. In the data processing stage, the Seurat analysis tool was used to conduct principal component analysis and implement cell clustering. Through dimensionality reduction techniques, including t‐SNE and UMAP methods, a visualized cell distribution map is generated. Subtype identification of each cell cluster was performed using the Celldex tool, and ultimately, all cells were annotated into 12 subgroups: T cells, CD4+ T cells, epithelial cells, B cells, macrophages, fibroblasts, endothelial cells, HSCS, monocytes, and neurons. On this basis, the expression data of EP300, LDHC, and STMN1 genes in different cell subpopulations were obtained, respectively, and visual expression analysis was conducted through dot plots and feature distribution plots. To further investigate the dynamic developmental transitions and intercellular communication networks within the UC mucosal microenvironment, we performed pseudotime trajectory analysis using monocle2 to reconstruct cellular differentiation lineages. Additionally, we employed CellChat to quantitatively infer and compare the intercellular communication patterns, identifying significantly altered signaling pathways between different cell types based on ligand–receptor expression profiles.

### 2.6. Cell Culture and Establishment of UC Type

Cell culture and establishment of the UC model: Colon mucosal epithelial cells (CP‐H040) were purchased from Pricella (confirmed to be free of mycoplasma contamination before use) and were cultured in RPMI 1640 (Gibco; 21870084) complete medium supplemented with 10% fetal bovine serum (FBS), 1% glutamine (Gibco; 25030081), and 1% penicillin–streptomycin (Gibco; 15070063) at 37°C and 5% CO_2_. LPS solution was dissolved in the above complete medium, and the LPS concentration was adjusted to 5 *μ*g/mL. When the cell density reached 80%, the cells were washed twice with PBS, and the PBS was discarded. Then, the cells were exposed to RPMI 1640 complete medium containing 5 *μ*g/mL LPS for 24 h. After the culture was completed, the cells were washed with PBS, and total RNA was extracted using RNA Isolater Total RNA Extraction Reagent (Vazyme; R401‐01).

### 2.7. qPCR Analysis of Markers Related to UC

Total RNA of cells was extracted using RNA Isolater Total RNA Extraction Reagent as per the instructions. Its purity and concentration were detected using an enzyme‐linked immunosorbent assay (ELISA) reader, and the sample concentration was uniformly adjusted. Reverse transcription was performed using the ABScript II cDNA First‐Strand Synthesis Kit (ABclonal, RK20400), and 2X Universal SYBR Green Fast qPCR Mix (ABclonal) for real‐time quantitative PCR (RK21203) was performed in a fluorescence quantitative PCR instrument (Bio‐Rad, CFX Connect). The reaction program is set as follows: predenaturation at 95°C for 30 s. Subsequently, 40 cycles were carried out, including denaturation at 95°C for 15 s and annealing/elongation at 60°C for 30 s. Taking GAPDH as the internal reference gene, the relative expression level of the target gene was calculated by the 2^−*ΔΔ*Ct^ method. All primers used in the study are listed in Supporting Information 2: Table S2.

### 2.8. Spatial Expression Characteristic Analysis

We downloaded the spatial transcriptome data of GSE189184 from the NCBI GEO database and selected two control groups (B10 and C5) and two disease groups (B4 and B8) for analysis. With the help of R packages such as Seurat and ggplot2, we conducted spatial transcriptome analysis on these four samples. After completing data preprocessing, dimensionality reduction clustering, and differential expression analysis, we focused on visualizing and analyzing the three core genes EP300, LDHC, and STMN1. We used SpatialDimPlot to display the distribution of cell clusters in the tissue space and SpatialFeaturePlot to present the expression intensity of specific genes at spatial locations. By comparing the spatial expression patterns of different genes, we revealed their potential roles in disease occurrence and development and related spatial heterogeneity characteristics.

## 3. Result

### 3.1. MR‐Identified Lactylation‐Related Genes Associated With the Risk of UC

The 46 lactated gene lists were intersected with the genes corresponding to all significant eQTLs in eQTLGen, and ultimately, 29 candidate genes that were both involved in lactated modification and had significant eQTL signals were obtained (Figure 2A). The MR analysis results based on eQTL data show that among the 29 candidate genes, EP300 (IVWOR = 1.12, *p* = 4.32 × 10^−3^), LDHC (IVWOR = 1.09, *p* = 9.87 × 10^−3^), SLC16A4 (IVWOR = 1.08, *p* = 0.022). The *p* values of the IVW method of STMN1 (IVW OR = 1.21, *p* = 3.15 × 10^−4^) were all less than 0.05, suggesting a potential causal relationship between gene expression levels and the risk of UC (Figure 2B). The pleiotropy test (MR‐Egger intercept *p* > 0.05, MR‐PRESSO global test *p* > 0.05) indicated that the results were less likely to be affected by horizontal pleiotropy. There was mild heterogeneity (*Q*_*p*val < 0.05) in the heterogeneity test, so the results of the random effects IVW model were mainly adopted. To assess the robustness of the causal estimates, we generated scatter plots, leave‐one‐out plots, and funnel plots for all four MR‐significant genes (EP300, LDHC, SLC16A4, and STMN1). As shown in Figure 2C–E (representative results for EP300, LDHC, SLC16A4, and STMN1), the scatter plots showed consistent directionality across different MR methods (Figure 2C). The leave‐one‐out analyses indicated that no single SNP disproportionately drove the causal estimates for any of the four genes (Figure 2D), and the funnel plots revealed no obvious asymmetry, suggesting minimal directional pleiotropy (Figure 2E).

Figure 2(A) The intersection of lactating genes and eQTL. (B) The results of the Mendelian randomization analysis of the four core genes. (C) Scatter plots for EP300, LDHC, SLC16A4, and STMN1. (D) Leave‐one‐out plots for EP300, LDHC, SLC16A4, and STMN1. (E) Funnel plots for EP300, LDHC, SLC16A4, and STMN1.

**Figure:**
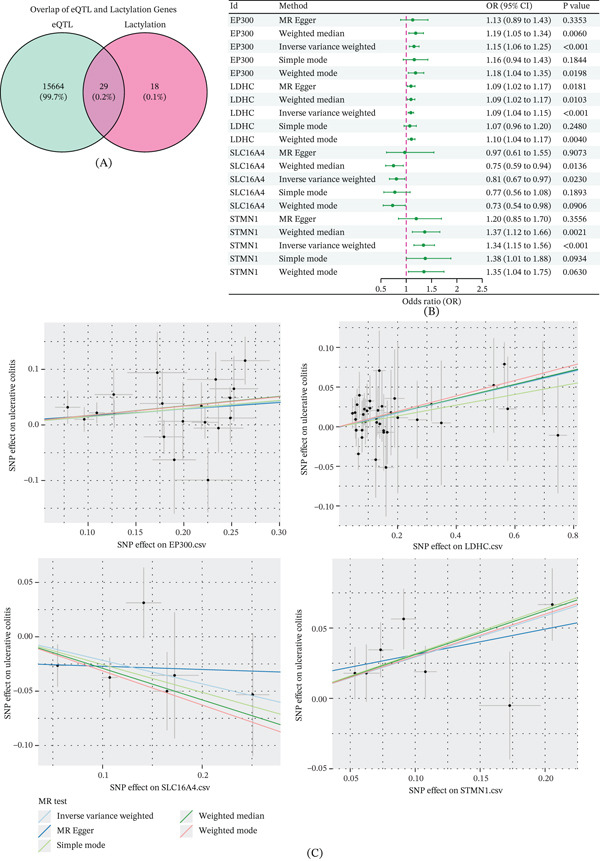
(A)

**Figure:**
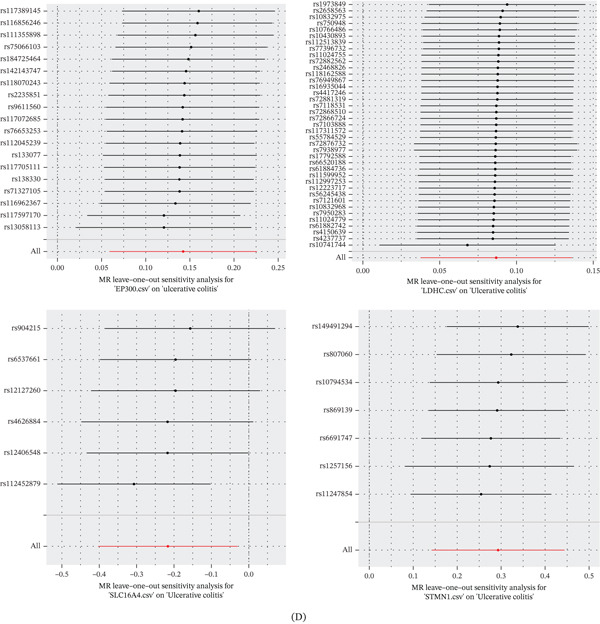
(B)

**Figure:**
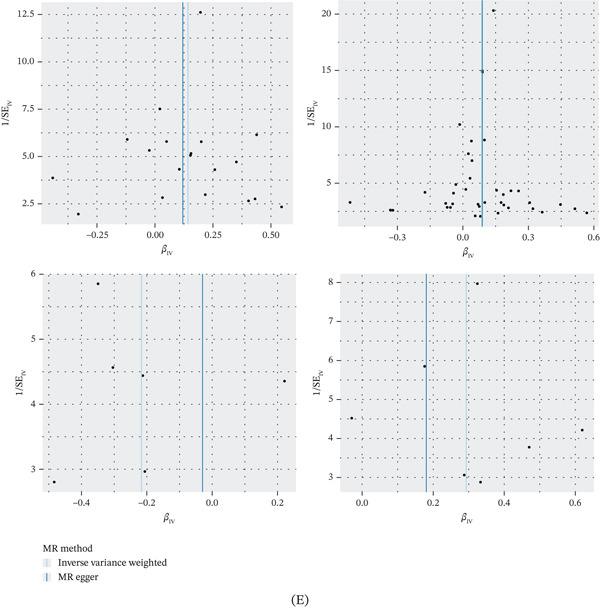
(C)

### 3.2. Result of SMR

SMR analysis further verified the significant association between STMN1 (*p*_SMR = 0.010, *p*_HEIDI = 0.153), LDHC (*p*_SMR = 0.013, *p*_HEIDI = 0.823), and EP300 (*p*_SMR = 0.014, *p*_HEIDI = 0.062) and UC, all passing the HEIDI test (*p*_HEIDI > 0.05), suggesting that these associations are not driven by LD. However, SLC16A4 (*p*_SMR = 0.347, *p*_HEIDI = 0.122) failed the verification (Table 1). Therefore, STMN1, LDHC, and EP300 were ultimately determined as the core genes for subsequent analysis. For detailed table content, please refer to Supporting Information 1: Table S1.

**Table 1:** Gene association verification results based on SMR analysis.

Gene	SNP	Position (chr:bp)	Allele (A1 > A2)	*b*_SMR	se_SMR	*p*_SMR	*p*_HEIDI	nsnp_HEIDI	OR (95% CI)
STMN1	rs1257156	1:26297984	G > T	0.326518	0.127501	0.01043969	0.1528809	20	1.38613319993606 (1.07962746303951–1.7796557736274)
SLC16A4	rs4626884	1:110938530	G > T	−0.213086	0.226439	0.3466898	0.1224338	20	0.808086638777164 (0.518452720821256–1.25952471564964)
LDHC	rs78454952	11:18438770	A > G	0.0920888	0.0370597	0.01295956	0.8225569	20	1.09646218359981 (1.01964220668554–1.17906978759974)
EP300	rs13058113	22:41508414	T > G	0.198396	0.0805509	0.01377839	0.06243086	20	1.21944519851862 (1.04134821689491–1.42800128531854)

### 3.3. Batch Effect Removal Following GEO Dataset Integration

**Figure 3:**
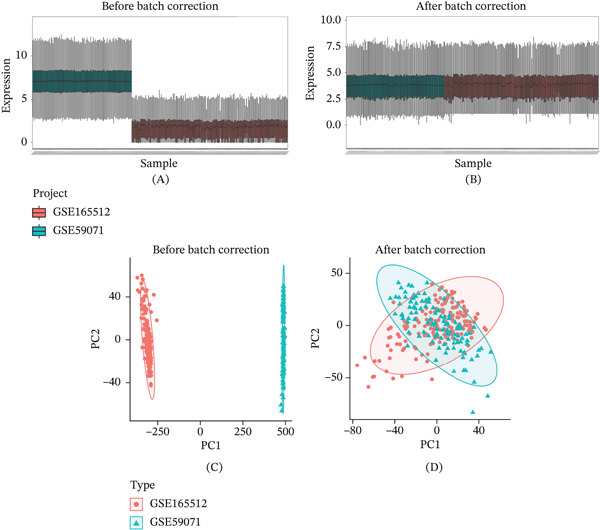
Union of dual datasets for ulcerative colitis. (A) Box plot of the integrated GSE59071 and GSE165512 datasets. (B) Box plot after normalization. (C) PCA clustering plot of normal and UC groups before batch correction. (D) PCA clustering plot of normal and UC groups after batch correction.

The box plot and PCA plot show that before correction, the GSE59071 and GSE165512 samples were significantly separated due to batch effects, indicating a strong batch effect. After processing with the ComBat function of the “sva” package, the median and interquartile range of the box plot tended to be consistent (as shown in Figure 3A,B). In the PCA plot, the two batches of samples overlapped in the PC1–PC2 space (as shown in Figure 3C,D), and the differences between batches disappeared, whereas the differences between disease and normal within the group were retained, indicating that batch correction was effective and biological differences were not lost.

### 3.4. Expression of Core Genes in UC Tissues

**Figure 4:**
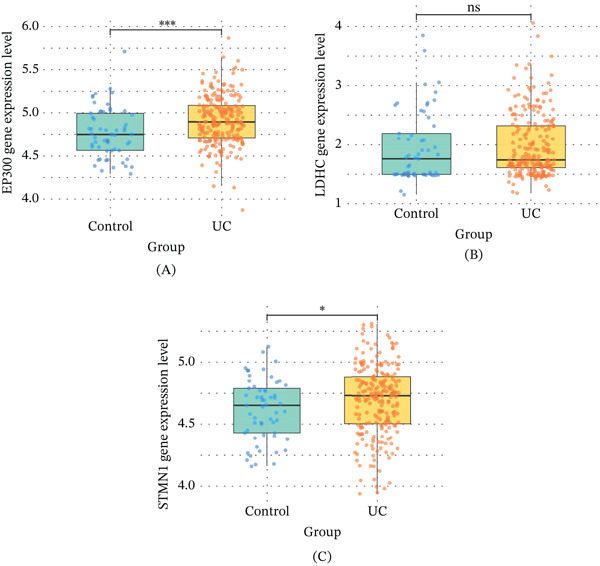
(A–C) Hub gene expression comparison in the GSE59071 and GSE165512 datasets between normal and UC groups. ns indicates *p* > 0.05,  ^∗^ *p* < 0.05,  ^∗∗∗^ *p* < 0.001.

Validation analysis based on the GEO datasets (GSE59071 and GSE165512) indicated that, compared with the healthy control group, the expression levels of EP300 and STMN1 genes in the colonic mucosa tissue of UC patients were significantly upregulated. The expression alterations of these genes suggest that they play a key role in disease progression and may serve as potential biomarkers or therapeutic targets (Figure 4).

### 3.5. Single‐Cell Analysis of Cell‐Specific Expression of Core Genes

Single‐cell transcriptome analysis successfully resolved the cellular heterogeneity in UC colon tissue. The UMAP dimensionality reduction map clearly displayed 12 well‐annotated cell subpopulations, including B cells, CD4+ T cells, epithelial cells, endothelial cells, and macrophages (Figure 5A,B). Expression visualization analysis revealed that the three genes exhibited obvious cell specificity (Figure 5C,D): STMN1 was highly expressed in B cells and monocytes, whereas LDHC and EP300 were significantly enriched in endothelial cells, suggesting their potential involvement in modulating specific immune or stromal cell functions during UC pathogenesis.

Figure 5Expression profiles of hub genes in single cells. (A) Cellular subtypes of UC. (B) A diagram showing the distribution ratio of 12 types of cells. (C, D) Scatter plots and a bubble plot of the expression of the four hub genes. (E) Trajectories and proposed time sequences of 12 types of cells. (F) Intercellular communication networks in UC identified by CellChat.

**Figure:**
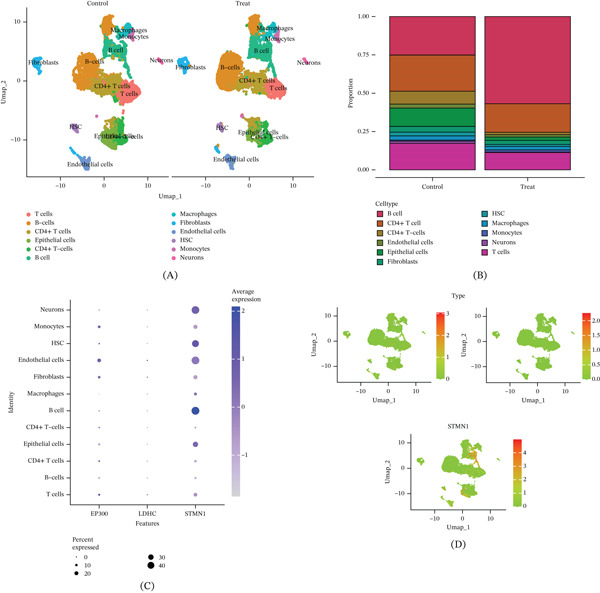
(A)

**Figure:**
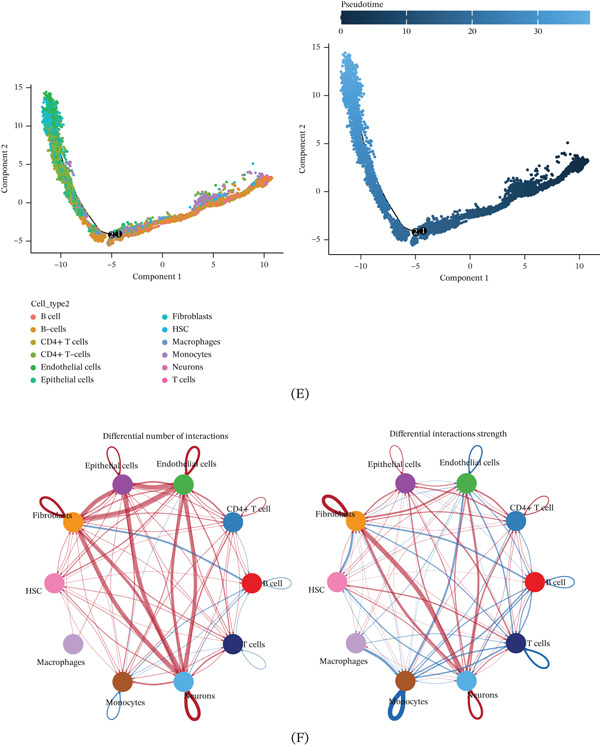
(B)

To delineate the developmental dynamics of these cell populations, we performed pseudotime trajectory analysis. The trajectory plot revealed a continuous differentiation landscape, with epithelial cells, fibroblasts, and endothelial cells distributed along distinct branches, suggesting potential lineage commitment and transitional states in the inflammatory microenvironment (Figure 5E).

Furthermore, we systematically characterized intercellular communication networks using CellChat. The analysis of the differential number of interactions and interaction strength among cell types revealed that B cells and endothelial cells serve as central hubs in the UC inflammatory niche, exhibiting markedly increased outgoing and incoming signaling compared with other populations (Figure 5F). These findings indicate that lactylation‐related genes, particularly STMN1 in B cells and EP300 and LDHC in endothelial cells, may contribute to UC pathogenesis by orchestrating immune‐stromal crosstalk and maintaining proinflammatory signaling loops.

### 3.6. qPCR Verification of Core Gene Expression

**Figure 6:**
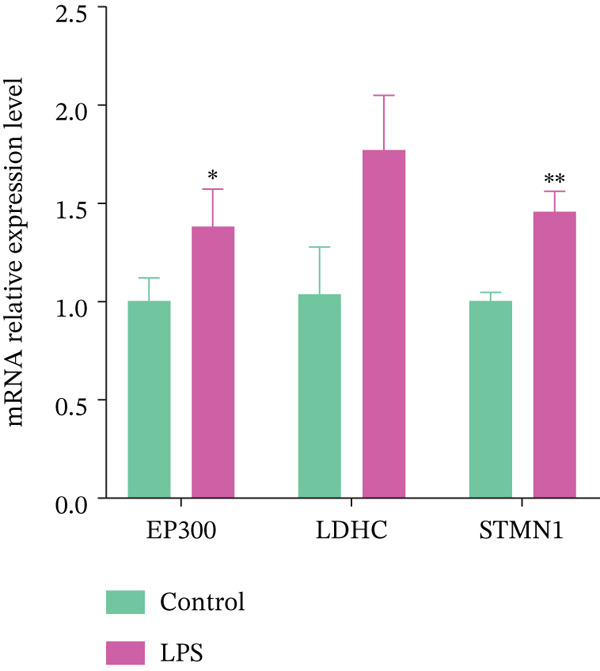
The qPCR analysis results of key gene expression.  ^∗^ *p* < 0.05,  ^∗∗^ *p* < 0.01.

The expression levels of core immune genes (including STMN1, LDHC, and EP300) in the UC model were detected using fluorescence quantitative PCR technology. The experimental results showed that compared with the normal control group, the expression levels of STMN1 and EP300 in the LPS group were both increased, and there was a significant difference compared with the normal group. This provides guidance for the diagnosis and treatment of UC. Additionally, for LDHC, *p* > 0.05; therefore, the expression difference between groups was not statistically significant (Figure 6).

### 3.7. Spatial Expression Characteristics and Disease‐Related Changes of Key Genes

**Figure 7:**
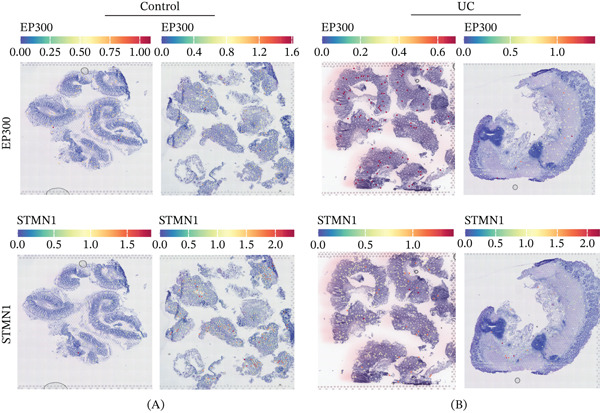
The expression levels of key genes in the sample. (A, B) Scatter plots show the expression levels of key genes in spatial transcriptomic samples. (A) Control group; (B) experimental group.

We analyzed spatial transcriptomic data to assess the expression levels of two key genes (due to sample data reasons, the LDHC gene was not expressed in the spatial transcriptomic data and thus is not presented or discussed). Figure 7 shows the spatial expression of the EP300 and STMN1 genes in the sample. This graph shows the expression levels of genes in these samples through color gradients, with darker colors indicating higher expression levels. Compared with the control group, EP300 and STMN1 were significantly upregulated in the disease group.

## 4. Discussion

UC is a chronic idiopathic inflammatory bowel disease characterized by continuous inflammation of the colonic mucosa, usually starting from the rectum and extending toward the proximal end [15]. Clinically, patients often present with bloody diarrhea, abdominal pain, a sense of urgency, and tenesmus. Some patients also have systemic symptoms such as weight loss and fatigue [16, 17]. UC can lead to a variety of serious problems. Severe postcolectomy enteritis associated with UC may occur, presenting as massive intestinal bleeding, intestinal perforation, or high‐output stoma, and may be complicated by hypovolemic shock or disseminated intravascular coagulation, with a mortality rate as high as 11.9% [18]. In recent years, the current epidemiological characteristics of UC are characterized by a continuous increase in global incidence and prevalence [19, 20] and a common bimodal age distribution (peaks in youth and old age) as well as regional gender differences [21, 22]. At present, endoscopic examination and biopsy are the only methods for diagnosing UC [23]. There are various treatment methods for this disease. In clinical practice, drug therapy is mainly relied upon, such as 5‐aminosalicylic acid (5‐ASA), corticosteroids, immunosuppressants, and biologics. However, these drugs have limitations, including side effects, individual differences, compliance issues, and economic burdens. There is no ideal solution at present. Therefore, it is particularly urgent to find a better treatment plan.

Lactylation is a newly discovered PTM that involves the covalent binding of lactic acid molecules to the lysine residues of proteins, thereby regulating protein function and gene expression [24, 25]. Lactylation not only is a metabolic product of glycolysis but also participates in epigenetic regulation as a signaling molecule [26]. The occurrence of lactylation is closely related to cellular metabolic reprogramming, especially under the condition of enhanced glycolysis, where a large amount of lactic acid accumulates, providing a substrate for lactylation. In addition, lactylation modulates processes such as transcription factor activity, inflammatory response, and cell proliferation by altering the spatial conformation of proteins [3, 24, 27].

In terms of diseases, lactylation has been proven to play a key role in a variety of pathological conditions. In tumors, lactylation can promote tumor cell proliferation, metastasis, and immunosuppression, for instance, by upregulating oncogenic signaling pathways or inducing polarization of M2‐type macrophages [24, 28, 29]. In cardiovascular diseases, lactylation is involved in the regulation of myocardial fibrosis and heart failure [27], whereas in obstetric diseases such as preeclampsia, abnormal lactylation levels in placental tissue are associated with energy metabolism disorders [30]. In addition, lactylation also includes nonhistone modifications, thereby expanding its potential functions in metabolic pathways such as the TCA cycle.

Unlike previous studies that screened for UC diagnostic markers based on machine learning [5], this study focused on the genetic causal inference of lactylation modification. It employed MR to effectively avoid confounding biases in traditional observational studies and combined single‐cell transcriptomics, spatial transcriptomics, and in vitro experiments for multidimensional validation, thereby providing a new methodological perspective and evidence level for the etiological research of UC.

In recent years, studies have shown that lactylation not only is limited to fields such as oncology but also plays a key role in various inflammatory and metabolic diseases. For instance, lactylation influences gene transcription, immune cell function, and inflammatory response by regulating the modification of histones and nonhistones. In addition, lactylation is closely related to glycolytic metabolism, and metabolic reprogramming is a common feature of many inflammatory diseases [3, 25]. In UC, the intestinal mucosa is often in a chronic inflammatory state, leading to local hypoxia and abnormal energy metabolism, which may promote the enhancement of glycolytic pathways and lactic acid accumulation [30]. Lactylation can also regulate mitochondrial function and TCA cycle–related proteins, influencing energy metabolism and oxidative stress responses [30, 31]. In UC, mitochondrial dysfunction and reactive oxygen species (ROS) accumulation are common pathological features, and lactylation may exacerbate intestinal inflammation by interfering with these metabolic pathways. Studies have shown that the lactic acid level in the intestinal tract of UC patients is significantly elevated, especially during the active stage of the disease. The lactic acid concentration in feces is positively correlated with the severity of inflammation [32, 33]. This lactic acid–rich environment promotes histone lactylation, which in turn regulates macrophage polarization toward the proinflammatory M1 phenotype and exacerbates the intestinal inflammatory response [34]. In addition, intestinal flora disorders (such as an increase in *Enterococcus* and a decrease in *Bifidobacterium*) may produce excessive lactic acid through the metabolism of mucus and hyaluronic acid, further driving the lactylation process [35]. Targeting the lactylation pathway (such as using the traditional Chinese medicine compound Gegen Qinlian decoction) can alleviate colitis [36] and oxidative stress by inhibiting lactic acid production and histone lactylation modification, suggesting that lactylation may be a potential target for the treatment of UC [34].

This study linked lactic acid modification to the genetic susceptibility of UC by systematically integrating multiomics data. Through MR analysis, it is suggested that the elevated expression levels of STMN1, LDHC, and EP300 genes may be causal risk factors for the onset of UC. Subsequently, we verified the abnormal expression of these genes at the tissue level and single‐cell level and ultimately confirmed their responses to inflammatory stimuli through in vitro experiments. Of particular note, STMN1 showed the strongest correlation signal and the most significant upregulation across all analyses. STMN1 is a microtubule‐unstable protein that participates in the regulation of cell mitosis and is usually highly expressed in various malignant tumors. In the context of UC, long‐term inflammation can significantly increase the risk of dysplasia and canceration of the mucous membrane. Research has found that STMN1 specifically accumulates in colorectal dysplasia and tumor lesions of UC patients. Notably, traditional markers such as p53 are not expressed in some lesions, suggesting that STMN1 may have higher sensitivity [37]. In addition, our single‐cell analysis found that it was highly expressed in B cells and epithelial cells, suggesting that it might be involved in the occurrence and development of UC by regulating immune cell activation or the barrier function and repair of epithelial cells.

The p300 protein encoded by the EP300 gene is a transcriptional coactivator that is widely involved in the regulation of cellular signaling pathways, including the WNT pathway, which plays a significant role in the proliferation, differentiation, and immune regulation of intestinal epithelial cells. In UC, chronic inflammation is the core feature, involving the abnormal activation of various immune cells and inflammatory factors. Studies have found that EP300 harbors mutations more frequently or uniquely in IBD‐related colorectal tumors, suggesting that genetic alterations in EP300 may be associated with the inflammation‐driven process of UC [38]. For instance, EP300 mutations may exacerbate inflammatory responses by disrupting the WNT signaling pathway, affecting the barrier function and immune homeostasis of the intestinal epithelium.

Single‐cell analysis revealed that lactylation‐related genes were highly expressed in endothelial cells and B cells. In the pathogenesis of UC, endothelial cells play a key role, and their functional abnormalities and activation status are closely related to the activity of the disease and the inflammatory process. Firstly, endothelial cells are significantly activated in UC, as shown by the increased activity of nitric oxide synthase (NOS). Studies have shown that microvascular endothelial cells isolated from the mesentery of UC patients exhibit high NOS activity, whereas no similar phenomenon has been detected in healthy controls or colon cancer patients [39]. This high activity is mainly attributed to the expression of inducible NOS (iNOS), indicating that endothelial cells are in a continuously activated state in UC, which may be related to local vascular integrity disruption and inflammation maintenance [40]. Secondly, the upregulation of adhesion molecule expression on the surface of endothelial cells is an important feature in the inflammatory process of UC. In UC patients, the expressions of ICAM‐1, VCAM‐1, and E‐selectin on endothelial cells are significantly increased. These molecules promote the adhesion of lymphocytes and macrophages to endothelial cells, thereby maintaining chronic inflammation [41]. Abnormal activation of B cells is associated with the inflammatory progression of UC. The B cells in the lamina propria of the intestinal mucosa of UC patients show a highly activated state. The expressions of surface markers CD71, CD25, and 4F2 are significantly increased, and there is a transition phenomenon from CD45RA to CD45RO, suggesting that B cells are changing from the resting state to the activated state [42]. In addition, B cells derived from the appendix can migrate to the colon via the CCL20–CCR6 axis. This migration promotes the differentiation of CD4+ T cells into Th1/Th17 cells, exacerbating colonic inflammation [43].

However, this article still has certain limitations. Specifically, the spatial transcriptomic analysis was based on a limited number of samples (two controls and two UC samples) from the GSE189184 dataset. This small sample size may limit statistical power and increase the risk of sampling bias, potentially leading to false negatives or unstable findings. The spatial expression patterns of STMN1 and EP300 should be considered exploratory and require validation in larger independent spatial transcriptomic cohorts. In the future, expanding the tissue sample size can enhance the robustness of the association between lactic acid genes and UC. Although potential targets have been screened out, there is a lack of functional experiments that directly intervene in lactic acid modification, and the causal evidence is still incomplete. In addition, the research conclusions are mainly based on GWAS in European and American populations. Caution is needed when extrapolating them to other ethnic groups. Furthermore, our primary exposure data were derived from whole blood eQTLs. Given that UC is a colonic inflammatory disease, gene expression regulation in the target tissue may differ. Although we observed consistent eQTL effects for our core genes in colon tissue from the GTEx database, this cannot fully eliminate the potential bias. Future studies using colonic mucosa–specific eQTLs are warranted to validate our findings. Therefore, only by conducting multicenter and multiethnic in vivo and in vitro functional validations and prospective cohorts can the true pathogenicity and clinical translational value of these lactic acid targets be further confirmed.

## 5. Conclusion

In summary, this study, by integrating MR, multiomics data, and experimental verification, systematically revealed the potential causal roles of lactylation‐related genes STMN1, LDHC, and EP300 in the pathogenesis of UC. STMN1 demonstrated the strongest consistency in genetic association, tissue expression, single‐cell mapping, and inflammatory models, suggesting that it is not only a potential risk gene for UC but also likely a key molecular node connecting chronic inflammation and cancerous transformation. LDHC and EP300 are, respectively, involved in immune microenvironment imbalance and epithelial barrier dysfunction by regulating glycolytic metabolism and transcriptional activation. This study provides new molecular markers and therapeutic targets for UC and also lays a theoretical foundation for subsequent multicenter and multipopulation functional validation and clinical translation.

## Author Contributions

N.A.: study design, data acquisition, and manuscript drafting; R.W.: manuscript drafting, data analysis, and interpretation; H.J.: statistical analysis, methodology validation, and manuscript revision; Z.L.: data curation, technical support, and literature review; Y.D.: investigation support and critical feedback on results; L.Z.: resources, supervision, and project administration; Z.D.: conceptualization, funding acquisition, supervision, and final approval of the manuscript. N.A., R.W., and H.J. contributed equally to this work.

## Funding

The authors declare that financial support was received for the research and/or publication of this article. The study was funded by Rizhao City Traditional Chinese Medicine Science and Technology Project (RZY2025A02) and 2025 National Integrated Traditional Chinese Medicine Reform Demonstration Zone Science and Technology Co‐construction Project.

## Ethics Statement

The authors have nothing to report.

## Consent

The authors have nothing to report.

## Conflicts of Interest

The authors declare no conflicts of interest.

## Supporting Information

Additional supporting information can be found online in the Supporting Information section.

## Supporting information


**Supporting Information 1** Table S1: The SMR analysis results in detail.

**Supporting Information 2** Table S2: qPCR primer sequences of the experiments.

**Supporting Information 3** File S1: STROBE‐MR report list.

## Data Availability

The data that support the findings of this study are available in the GEO database, eQTLGen consortium, and OpenGWAS at https://www.ncbi.nlm.nih.gov/geo/ (Reference Numbers GSE59071, GSE165512, GSE231993, and GSE189184; finngen_R12_U). These data were derived from the following resources available in the public domain: GEO database (https://www.ncbi.nlm.nih.gov/geo/), eQTLGen consortium (https://www.eqtlgen.org), and OpenGWAS (https://opengwas.io/).
