# Supplementary material for: Lactylation‐Related Genes in Ulcerative Colitis: A Multiomics Mendelian Randomization Study for Therapeutic Target Discovery
Source: Hum Mutat. 2026 Jun 26;2026:7720538. doi: 10.1155/humu/7720538 (PMC13307184; doi:10.1155/humu/7720538)
Supplement: Supplementary file 3 — Supporting Information 3 File S1: STROBE‐MR report list. [file HUMU-2026-7720538-s003.docx]

**STROBE-MR checklist of recommended items to address in reports of Mendelian randomization studies**^1^ ^2^

| **Item No.** | **Section** | **Checklist item** | **Page No.** | **Relevant text from manuscript** |
| --- | --- | --- | --- | --- |
| 1 | **TITLE and ABSTRACT** | Indicate Mendelian randomization (MR) as the study’s design in the title and/or the abstract if that is a main purpose of the study | 1 | Title：Integrative multi-omics summary-based mendelian randomization...；The abstract mentions “Mendelian randomization identified...” |
|  | **INTRODUCTION** |  |  |  |
| 2 | **Background** | Explain the scientific background and rationale for the reported study. What is the exposure? Is a potential causal relationship between exposure and outcome plausible? Justify why MR is a helpful method to address the study question | 2 | The introduction section provides a detailed explanation of the pathogenesis of UC, the role of lactoylation, and the reason for using the MR method (“to avoid the confounding bias in traditional observational studies”) |
| 3 | **Objectives** | State specific objectives clearly, including pre-specified causal hypotheses (if any). State that MR is a method that, under specific assumptions, intends to estimate causal effects | 2 | The last paragraph of the introduction clearly states the research objective: "…using Mendelian randomization... to screen for lactoylation-related genes that may have a causal relationship with UC" |
|  | **METHODS** |  |  |  |
| 4 | **Study design and data sources** | Present key elements of the study design early in the article. Consider including a table listing sources of data for all phases of the study. For each data source contributing to the analysis, describe the following: | 3-4 | The Method section clearly specifies the data sources (such as eQTLGen, FinnGen, GEO datasets, etc.), and describes the sample sources, selection criteria, sample size, etc. |
|  | a) | Setting: Describe the study design and the underlying population, if possible. Describe the setting, locations, and relevant dates, including periods of recruitment, exposure, follow-up, and data collection, when available. | 3-4 | eQTLGen (whole blood), FinnGen (Finnish population), and GEO (public dataset) are all described in the methods section. |
|  | b) | Participants: Give the eligibility criteria, and the sources and methods of selection of participants. Report the sample size, and whether any power or sample size calculations were carried out prior to the main analysis | 3-4 | eQTLGen (with over 30,000 samples), FinnGen (482,657 participants), GEO datasets (GSE59071, GSE165512) |
|  | c) | Describe measurement, quality control and selection of genetic variants | 3-4 | “SNPs that were significantly correlated with gene expression (P < 5×10⁻⁸) and independent of each other...” |
|  | d) | For each exposure, outcome, and other relevant variables, describe methods of assessment and diagnostic criteria for diseases | 3 | The exposure was related to the expression of lactoylation-related genes, and the outcome was UC (from the FinnGen database). The diagnostic criteria and variable processing methods were described in the method. |
|  | e) | Provide details of ethics committee approval and participant informed consent, if relevant |  | 未涉及 |
| 5 | **Assumptions** | Explicitly state the three core IV assumptions for the main analysis (relevance, independence and exclusion restriction) as well assumptions for any additional or sensitivity analysis | 3-4 | “This study conducted Mendelian randomization analysis based on three core assumptions: the correlation assumption... and the exclusion restriction assumption, which stipulates that the SNPs only influence the outcome (UC) through their effect on the exposure, with no other pathways involved....SNPS that were significantly correlated with gene expression (P < 5×10⁻⁸) and independent of each other (clumping r² < 0.001)... To evaluate the reliability... used the MR-Egger intercept test and the MR-PRESSO global test (Pleiotropy RESidual Sum and Outlier).” |
| 6 | **Statistical methods: main analysis** | Describe statistical methods and statistics used | 3-4 | Detailed explanations of five MR methods (IVW, MR-Egger, Weighted Median, etc.), Cochran's Q test, MR-PRESSO, etc. |
|  | a) | Describe how quantitative variables were handled in the analyses (i.e., scale, units, model) |  | It is not explicitly stated, but gene expression is a continuous variable, and the outcome is binary (UC vs. control) |
|  | b) | Describe how genetic variants were handled in the analyses and, if applicable, how their weights were selected | 3 | SNPs selection criteria：P < 5×10⁻⁸，clumping r² < 0.001，window size = 10,000 kb |
|  | c) | Describe the MR estimator (e.g. two-stage least squares, Wald ratio) and related statistics. Detail the included covariates and, in case of two-sample MR, whether the same covariate set was used for adjustment in the two samples | 3-4 | Mainly using the IVW method, no specific mention was made of covariate adjustment |
|  | d) | Explain how missing data were addressed |  | Not mentioned |
|  | e) | If applicable, indicate how multiple testing was addressed |  | No multiple testing correction was mentioned. |
| 7 | **Assessment of assumptions** | Describe any methods or prior knowledge used to assess the assumptions or justify their validity | 3-4 | The heterogeneity was evaluated using Cochran's Q test, while the level pleiotropy was assessed by MR-Egger intercept test and MR-PRESSO. |
| 8 | **Sensitivity analyses and additional analyses** | Describe any sensitivity analyses or additional analyses performed (e.g. comparison of effect estimates from different approaches, independent replication, bias analytic techniques, validation of instruments, simulations) | 3-4 | Use MR-Egger, Weighted Median, Simple Mode, and Weighted Mode for sensitivity analysis, and mention the "leave-one-out" method. |
| 9 | **Software and pre-registration** |  |  |  |
|  | a) | Name statistical software and package(s), including version and settings used | 3 | Mainly using R version 4.5.0, packages such as "TwoSampleMR", "pacman", and "ieugwasr" etc. |
|  | b) | State whether the study protocol and details were pre-registered (as well as when and where) |  | Not applicable |
|  | **RESULTS** |  |  |  |
| 10 | **Descriptive data** |  |  |  |
|  | a) | Report the numbers of individuals at each stage of included studies and reasons for exclusion. Consider use of a flow diagram | 6-7 | The intersection of 46 lactoylation genes and eQTLGen → 29 candidate genes → 4 core genes were selected through MR analysis → 3 final genes were determined after SMR verification. |
|  | b) | Report summary statistics for phenotypic exposure(s), outcome(s), and other relevant variables (e.g. means, SDs, proportions) | 6-13 | Figure 2-7 presents the differences in gene expression, the results of single-cell clustering, and the spatial expression patterns, etc. Table 1 shows the detailed statistics of the SMR analysis. |
|  | c) | If the data sources include meta-analyses of previous studies, provide the assessments of heterogeneity across these studies |  | Not covered |
|  | d) | For two-sample MR:  i.  Provide justification of the similarity of the genetic variant-exposure associations between the exposure and outcome samples  ii.  Provide information on the number of individuals who overlap between the exposure and outcome studies | 4 | This study employed a two-sample Mendelian randomization design. The exposure data (eQTL) were obtained from the eQTLGen consortium with a predominantly European ancestry, while the outcome data (UC GWAS) were from the FinnGen database of the Finnish population. Although the two samples were independent, they both mainly originated from the European population, and their genetic backgrounds were comparable, which reduced the risk of bias caused by population stratification. We noticed that there might be some unknown overlap between the two samples, so we mainly used the random effects IVW model, which was relatively robust to sample overlap. |
| 11 | **Main results** |  |  |  |
|  | a) | Report the associations between genetic variant and exposure, and between genetic variant and outcome, preferably on an interpretable scale | 6 | Figure 2B presents the IVW OR and P values for each gene. |
|  | b) | Report MR estimates of the relationship between exposure and outcome, and the measures of uncertainty from the MR analysis, on an interpretable scale, such as odds ratio or relative risk per SD difference | 6-8 | Table 1 presents b_SMR, se_SMR, P value and OR (95% CI) for SMR |
|  | c) | If relevant, consider translating estimates of relative risk into absolute risk for a meaningful time period |  | Not mentioned |
|  | d) | Consider plots to visualize results (e.g. forest plot, scatterplot of associations between genetic variants and outcome versus between genetic variants and exposure) | 6-13 | Figures 2, 3, 4, 5, etc. present the results. |
| 12 | **Assessment of assumptions** |  | 6-13 | The results of the heterogeneity test (Cochran's Q) and the multiplicity test (MR-Egger, MR-PRESSO) indicate |
|  | a) | Report the assessment of the validity of the assumptions | 6 | “The pleiotropy Test (MR-Egger intercept P > 0.05, MR-PRESSO Global Test P > 0.05) indicated that the results were less likely to be affected by horizontal pleiotropy.” |
|  | b) | Report any additional statistics (e.g., assessments of heterogeneity across genetic variants, such as *I^2^*, Q statistic or E-value) | 6 | “There was mild heterogeneity (Q_pval < 0.05) in the heterogeneity test, so the results of the random Effects IVW model were mainly adopted.” |
| 13 | **Sensitivity analyses and additional analyses** |  | 6-7 |  |
|  | a) | Report any sensitivity analyses to assess the robustness of the main results to violations of the assumptions | 6 | The results of heterogeneity and multiplicity tests are presented above. The article mentions that multiple MR methods (such as MR-Egger, etc.) were used to compare with the IVW results. |
|  | b) | Report results from other sensitivity analyses or additional analyses | 6-7 | The SMR analysis further confirmed the association of the three genes (STMN1, LDHC, EP300) with UC, while ruling out SLC16A4. |
|  | c) | Report any assessment of direction of causal relationship (e.g., bidirectional MR) |  | No reverse MR or other directional tests were conducted. |
|  | d) | When relevant, report and compare with estimates from non-MR analyses |  | Not mentioned |
|  | e) | Consider additional plots to visualize results (e.g., leave-one-out analyses) |  | Not mentioned |
|  | **DISCUSSION** |  |  |  |
| 14 | **Key results** | Summarize key results with reference to study objectives | 13-14 | The first paragraph of the Discussion and the conclusion section summarize the key findings: "STMN1 and EP300 have been identified as the causal genes for UC." And the multi-dimensional verification results are reviewed. |
| 15 | **Limitations** | Discuss limitations of the study, taking into account the validity of the IV assumptions, other sources of potential bias, and imprecision. Discuss both direction and magnitude of any potential bias and any efforts to address them | 15 | The second to last paragraph of the discussion clearly states: "This article still has certain limitations." These include the sample size, the lack of direct functional experiments, and the need for caution when extrapolating to the population. |
| 16 | **Interpretation** |  |  |  |
|  | a) | Meaning: Give a cautious overall interpretation of results in the context of their limitations and in comparison with other studies | 13-17 | The discussion section carefully interprets the results and cites relevant literature to support them. |
|  | b) | Mechanism: Discuss underlying biological mechanisms that could drive a potential causal relationship between the investigated exposure and the outcome, and whether the gene-environment equivalence assumption is reasonable. Use causal language carefully, clarifying that IV estimates may provide causal effects only under certain assumptions | 15-16 | The potential role of STMN1 in the carcinogenesis of UC was discussed, the influence of EP300 on inflammation through the WNT pathway was examined, and the role of lactoylation in immune metabolism was explored. |
|  | c) | Clinical relevance: Discuss whether the results have clinical or public policy relevance, and to what extent they inform effect sizes of possible interventions | 17 | Propose“This study provides new molecular markers and therapeutic targets for UC, and also lays a theoretical foundation for subsequent multi-center and multi-population functional validation and clinical translation” |
| 17 | **Generalizability** | Discuss the generalizability of the study results (a) to other populations, (b) across other exposure periods/timings, and (c) across other levels of exposure | 16 | It was mentioned during the discussion that "the research conclusions are mainly based on GWAS of the European and American populations, and caution should be exercised when applying them to other ethnic groups." |
|  | **OTHER INFORMATION** |  |  |  |
| 18 | **Funding** | Describe sources of funding and the role of funders in the present study and, if applicable, sources of funding for the databases and original study or studies on which the present study is based | 17 | The statement clearly states: "No funding support." |
| 19 | **Data and data sharing** | Provide the data used to perform all analyses or report where and how the data can be accessed, and reference these sources in the article. Provide the statistical code needed to reproduce the results in the article, or report whether the code is publicly accessible and if so, where | 17 | All the publicly available data used in this study can be obtained from the following databases:  eQTL data is from the eQTLGen Consortium (https://www.eqtlgen.org/)  GWAS data is from the FinnGen Consortium (version R9)  Transcriptome data is from GEO (GSE59071, GSE165512, GSE231993, GSE189184) |
| 20 | **Conflicts of Interest** | All authors should declare all potential conflicts of interest | 17 | The statement explains“The authors declare no competing interests.” |

This checklist is copyrighted by the Equator Network under the Creative Commons Attribution 3.0 Unported (CC BY 3.0) license.

1. Skrivankova VW, Richmond RC, Woolf BAR, Yarmolinsky J, Davies NM, Swanson SA, et al. Strengthening the Reporting of Observational Studies in Epidemiology using Mendelian Randomization (STROBE-MR) Statement. JAMA. 2021;under review.

2. Skrivankova VW, Richmond RC, Woolf BAR, Davies NM, Swanson SA, VanderWeele TJ, et al. Strengthening the Reporting of Observational Studies in Epidemiology using Mendelian Randomisation (STROBE-MR): Explanation and Elaboration. BMJ. 2021;375:n2233.
